# The First Patient in Poland Treated for SMA with Nusinersen During Pregnancy

**DOI:** 10.3390/jcm13237366

**Published:** 2024-12-03

**Authors:** Elżbieta Stawicka, Urszula Skarżyńska, Agata Lipiec, Natalia Mazanowska, Joanna Stankiewicz, Monika Gos, Milena Wyszczelska-Namięta

**Affiliations:** 1 Clinic of Paediatric Neurology, Institute of Mother and Child, Kasprzaka 17A, 01-211 Warsaw, Poland; 2 Department of Organization and Accounts, Institute of Mother and Child, Kasprzaka 17A, 01-211 Warsaw, Poland; 3 Clinic of Obstetrics and Gynaecology, Institute of Mother and Child, Kasprzaka 17A, 01-211 Warsaw, Poland; 4 Clinic of Anaesthesiology and Intensive Care, Institute of Mother and Child, Kasprzaka 17A, 01-211 Warsaw, Poland; 5 Medical Genetics Department, Institute of Mother and Child, Kasprzaka 17A, 01-211 Warsaw, Poland; monika.gos@imid.med.pl

**Keywords:** neurology, neuromuscular disorders (NMD), pediatric, health economics, diagnosis, prenatal, disease-modifying therapy, spinal muscular atrophy (SMA), muscle weakness

## Abstract

**Background:** The accessibility of effective SMA (spinal muscular atrophy) treatment is resulting in a growing number of affected women reaching fertility age and deciding to conceive. Pregnancy in women with SMA is associated with a high risk of rapid progression of symptoms, including increased weakness, growing paresis, or even onset of respiratory failure requiring ventilation support. Muscle weakness frequently leads to disability, which in a high percentage is irreversible. Pre-term labor and delivery by cesarean section are the most commonly described cases in the literature. **Methods:** This paper aims to describe the first case in Europe, a 21-year-old patient treated with Nusinersen under the drug program during the third trimester of pregnancy. Despite the significant progression of the disease during pregnancy, the patient did not lose gait function. The pregnancy was ended at 33Hbd, and the baby’s condition was unremarkable. In addition to clinical data, this paper also discusses the economic aspects. **Conclusions:** Thanks to the rapid initiation of treatment, the patient did not lose her ability to walk, and a significant improvement in motor function was observed after the postpartum period. No side effects or negative effects on the fetus were observed.

## 1. Introduction

Spinal muscular atrophy is a disease of the lower motoneuron associated with progressive degeneration and loss of anterior horn cells in the spinal cord. Untreated patients develop progressive muscle weakness, initially of the proximal muscles of the lower limbs, then of the upper limbs, and ultimately of the respiratory muscles. Depending on the type of disease, symptoms may appear not only in early childhood, but also in adolescence or adulthood [1,2].

Pregnancy in a patients with SMA should be classified as high risk due to the possibility of premature delivery, miscarriage, intrauterine growth restriction (IUGR), and maternal back pain due to increasing pressure caused by abdominal enlargement [3].

Pregnancy in patients with SMA is associated with an exacerbation of disease symptoms between 31% and 42% of women [3]. Before the period of available treatment, a diagnosis of SMA was considered a contraindication to pregnancy [4]. The risk of complications from general anesthesia, such as prolonged respiratory failure while not being able to give birth by natural means due to abdominal and pelvic muscle weakness, were discouraging factors for both patients and physicians [4].

In Poland, SMA therapy is possible under the Ministry of Health’s drug program and is available to all patients, regardless of the type or severity of symptoms. However, until April 2024, pregnancy was an exclusion criterion for participation in drug programs. Patients who become pregnant had to discontinued treatment for this period, as well as for the duration of breastfeeding. As of April 2024, according to the favorable opinion of the EMA, continuation of treatment during pregnancy is permitted.

The aim of this publication is to present a case report of the first patient in Poland treated with Nusinersen during pregnancy.

## 2. Case Report

A 21-year-old female patient with a history of nicotinism in her 13th week of pregnancy was referred to the Genetic Outpatient Clinic of the Mother and Child Institute in Warsaw in November 2023. The reason was the diagnosis of SMA in her younger brother, who, at the age of about 16 years, manifested the symptoms of SMA: lower limb muscle weakness, leading to difficulty walking, poor balance, tremors (genetically confirmed: the presence of deletions of exons 7 and 8 in the SMN1 gene, four copies of the SMN2 gene). At that time, other family members were not tested.

The test results were received in December 2023. The patient was diagnosed with SMA type 3 due to the age of first symptoms under 20 years of life (homozygous deletion of exons 7 and 8 in the SMN1 gene, and homozygous duplication of exons 7 and 8 in the SMN2 gene).

It was also not possible to examine the baby’s father.

During the visit, the patient was introduced to the possibility of diagnostic testing, i.e., fetal amniocentesis or postnatal amniocentesis. However, she did not decide to undergo prenatal testing.

From the moment of the diagnosis, the patient received multispecialty coordinated care at the local hospital, including obstetrics-gynecology, cardiology, and physiotherapy, as well as continuous follow-up of her neurological condition at the Department of Child Neurology at the Child Neurology Clinic of the Mother and Child Institute.

From the anamnesis: The patient was born from the first pregnancy, with a body weight of 3750 g, and scored 10 points on the Apgar scale. The course of pregnancy was expected, the adaptation period was uncomplicated, and psychomotor development was correct. The patient’s clinical symptoms of SMA have been present for several years; it is difficult to determine the exact onset of symptoms. The patient said she never liked running and was regularly dismissed from physical education classes. For several years, lower limb strength has gradually weakened, especially proximally (fatigue with prolonged standing, problems with gym exercises). However, neither the patient nor her parents thought it might be a disease symptom, and they did not initiate any investigations.

About a month after the genetic diagnosis, the patient showed a worsening of SMA symptoms in the form of:

- Difficulty getting up from a sitting position;
- Walking upstairs while holding on to handrails;
- Inability to climb ladders;
- Intolerance of remaining standing for long periods;
- Complaints of generalized fatigue.

Due to the deterioration of motor function, efforts have been initiated to be able to start treatment with Nusinersen. Finally, the patient was qualified for treatment during pregnancy at the Department of Paediatric Neurology at the Mother and Child Institute in Warsaw after approval from the coordinating team for the treatment of SMA patients, the Ministry of Health, and the National Health Fund. The patient consented to the proposed treatment. Prior to the treatment, she was in fine general condition; in the neurological examination, deviations from the norm were found: the presence of fasciculation of the tongue and tremors in the upper limbs, while strength, tension, and deep reflexes were without deviations. In the lower extremities: significantly reduced muscle strength proximally > distally, the patient could not lift the lower extremities, positive Gower’s sign, no knee reflexes, Achilles tendon reflexes present bilaterally. Laboratory results showed microcytic anemia and slightly elevated CRP. She scored 44pct on the HFMSE scale. During her stay at the Department of Child and Adolescent Neurology of the Mother and Child Institute on 23 February 2024, the administration of the first dose of Nusinersen intrathecally was carried out, and the procedure carried on without complications, without symptoms of post-puncture syndrome. At the time of the first drug administration, she was in the 29th week of pregnancy.

After that, the patient received two consecutive doses of Nusinersen at 12 mg/5 mL, and procedures were passed without complications. During the above four weeks, significant neurological deterioration was observed: the patient reported significant fatigue and weakness in the lower extremities (she was able to walk a distance of about 50 m) and was unable to maintain a standing position for long periods. After a medical Consilium (including a gynecologist and neonatologist), the decision was made to deliver the pregnancy by elective cesarean section. The patient was transferred to the Department of Obstetrics and Gynecology. After anesthesiologic consultation, she was qualified for subarachnoid anesthesia (ASA III/IV). Steroid therapy (Bethametasone 25–26 March 2024) was administered to stimulate alveolar maturation. At 33 weeks gestation, a cesarean section was performed, and she gave birth to a live 2300 g/32 cm daughter in good general condition. The early postpartum period was uncomplicated. Currently, the patient moves for longer distances with the help of crutches and for shorter distances independently. The child currently does not require respiratory support and is trained to feed with a pacifier. The genetic test result for SMA is a heterozygous carrier, unaffected by SMA.

After the postpartum period, the patient was continuously rehabilitated, and a progressive improvement in motor function was observed, which is of particular importance in the context of the need to care for the infant. Five months after delivery, a 12-point improvement in the HSFME scale was achieved (62 points). Currently, the patient is able to move independently within her apartment, go for short walks, and do her work as a tattoo artist. The treatment with Nusinersen continues, and the patient is constantly followed up by the medical team and rehabilitated.

Total direct costs related to medical consultations and hospitalizations were at USD 15,796.37, indirect costs were at USD 937.74, and corporate overhead costs were at USD 1358.85 (at the average exchange rate of the National Bank of Poland for the U.S. dollar as of 16 April 2024: USD 4.0687) (Table 1). Revenues from the National Health Fund amounted to USD 4646.07, which must be added to the reimbursement for the purchase of the drug up to the amount of the purchase invoice. It is also worth noting that the public payer did not accept the financing based on the settlement catalog and, instead of financing the treatment at the Pediatric Neurology Clinic for USD 1691.22, paid USD 230.99. The two hospitalizations, during which the first and second doses were administered, amounted to a difference of USD 2920.45 between the financing and the primary settlement with the public payer. In addition, the Nusinersen therapy used generated costs at a selling price of USD 239,693.27, with the administration of three doses from 22 February to 24 March 2024. If the administrations were carried out as scheduled, the annual cost of the therapy would be USD 479,386.54 (six administrations from inclusion to December 2024).

It should be noted that this therapy involves hospitalization and lumbar puncture costs. The total cost of the patient’s referral from genetic testing to delivery was USD 262,432.30. After consultations, the public payer did not agree to fund the drug program, and the Mother and Child Institute obtained approval to fund the treatment under the Rescue Access to Drug Technology (RDTL).

**Figure 1:**
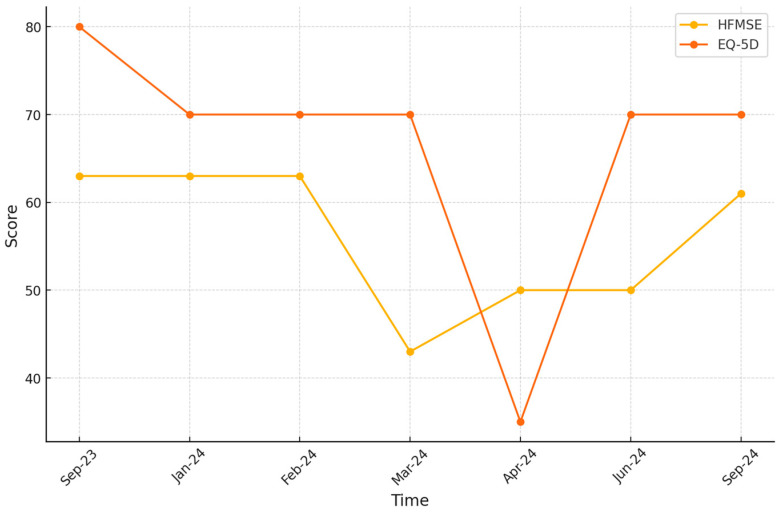
Patient’s score on HFMSE and EQ-5D scale over one year.

**Figure 2:**
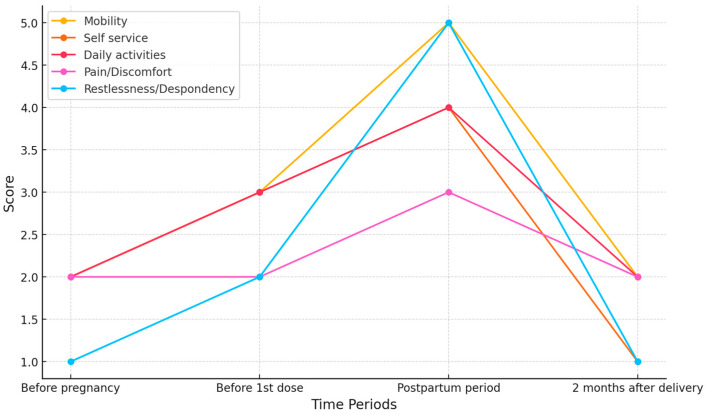
Patient’s score in EQ-5D scale.

Note the significant improvement in the patient’s quality of life after therapy, which correlates with her HFMSE scores (Figure 1). The EQ-5D scale shows a clear decline in the postpartum period and a significant improvement with the administration of subsequent doses of Nusinersen (Figure 2). A practical measurement is her ability to return to professional activity. Despite the initial loss of muscle strength that prevented her from working, the patient successfully returned to her tattoo artist profession. In Poland, income from tattoo art work ranges from USD 2950 to USD 9340 per month. By regaining the ability to work, the patient is now able to support her new family on her own, as well as finance her supplemental treatment, which is not funded by the payer. This highlights not only the clinical benefits, but also the economic impacts of the therapy.

## 3. Discussion

The number of available publications on this subject is considerably limited. The available descriptions are mainly of untreated patients, often presenting the milder form of SMA (type III, IV) [3,5]. The authors of most papers emphasize the differences in the management of pregnancy and delivery depending on whether the patient can move independently or in a wheelchair. In the latter, due to scoliosis and often more severe respiratory failure, the need for cesarean section under general anesthesia is more frequent. In walking patients, as in our patient, natural childbirth or regional anesthesia (spinal and epidural anesthetic techniques) is recommended [3,6].

Our patient’s case highlights the importance of conducting a comprehensive family history and genetic testing with the patient’s immediate family members. At the time of the genetic testing referral, the patient was not affected by any symptoms of SMA, while the rapid genetic diagnosis made it possible to implement dedicated treatment immediately.

To date, there has been no such description in the literature; in the available publications, the diagnosis of SMA was established before pregnancy.

In most studies, the decision to become pregnant was conscientious, and the patients were aware of the risks and complications. In this case, an important aspect was the patient’s mental state, helping her to accept her illness and growing disability. In one study [7], 74% of patients with SMA reported significant weakness during pregnancy, which did not improve after delivery in 42% of patients. So, there is a possibility that the functional status from the perinatal period will become enhanced in our patient.

The analysis of the costs associated with treating a patient with SMA type 3 during pregnancy shows that the management of therapy under the conditions of the Polish health system requires the meticulous consideration of many economic aspects.

The choice of Nusinersen treatment required a significant financial investment, reflected in the total direct costs of treatment and hospitalization. However, preserving the patient’s motor function indicates a potential clinical benefit that may outweigh the higher initial costs, especially regarding the long-term impact on quality of life.

A study [8] compared the costs of the two therapies with Nusinersen and Risdiplam, taking into account both the purchase price of the drug and the costs associated with its administration and overall treatment costs. While highlighting the lower cost of Risdiplam treatment, the authors also considered the aspect of long-term care and maintenance of patients’ quality of life. Comparing the cost of our patient’s Nusinersen therapy to an alternative therapy not used because of the qualifying criteria, Risdiplam, with a selling price (Ministry of Health notice) of USD 9456.39 per pack for a two-pack issue, would generate costs of USD 18,912.79 until delivery or USD 255,322.64 for a year of treatment.

## 4. Conclusions

This article describes a unique case of a young patient who became aware of her diagnosis of SMA type 3 in the second trimester of pregnancy. The patient showed courage and determination by agreeing to treatment as a first case in Poland. In addition, her trust in the doctors and good compliance resulted in the possibility of an early delivery, which preserved the mother’s gait function, as well as the well-being of the newborn.

The occurrence of pregnancy in SMA patients will be an increasing phenomenon due to the successful treatment of patients not only with type 3, but also with type 2 [5,6]. Therefore, there is a growing need to organize specialized centers to provide multi-specialist coordinated care for these patients [3]. Crucial is close cooperation between a neurologist, gynecologist, obstetrician, neonatologist, and anesthesiologist [6,9,10]. The experience of such centers will allow for further studies conducted on larger groups of patients to objectify the recommendations.

The development of a treatment strategy for a patient with SMA during pregnancy should be based on a thorough analysis of direct costs, indirect costs, and revenue opportunities from the National Health Fund, as well as considering the long-term health effects for mother and child. This will enable the efficient allocation of resources and optimization of treatment processes in the future.

Further research directions should include an evaluation of the effectiveness of treatment of women with SMA during pregnancy. The financial analysis results indicate the need for additional studies evaluating the long-term cost-effectiveness of Nusinersenum during pregnancy, comparing it to alternative therapies, mainly orally administered Risdiplam. Reducing the need for hospitalization could affect the overall economics of SMA treatment.

The results of the economic analysis indicate the need for an interdisciplinary approach to managing pregnancy and treating SMA, which may include medical and economic aspects. Therapeutic decision-making should consider not only the current cost of medications, but also the anticipated benefits and costs associated with the long-term care of the mother and child.

## Figures and Tables

**Table 1:** Analysis of healthcare costs of a patient with SMA type III during pregnancy.

Consultations			Direct Costs	Indirect Costs	Overhead Costs	Revenue from the National Health Fund (NFZ)	
From 9 November 2023 (Genetics Clinic) to 19 March 2024 (Obstetrics and Gynecology Clinic), there were 15 consultations with various specialists with the following costs:			USD 1016.41	USD 39.81	USD 15.25	USD 881.11	
Hospitalizations	Admission Date	Date of Discharge	Direct costs	Indirect costs	Overhead costs	Revenue from the National Health Fund (NFZ) excluding medicine.	Cost of medication as per the Minister of Health’s announcement
Obstetrics and Gynecology Clinic	20 February 2024	22 February 2024	USD 2762.56	USD 132.77	USD 188.07	USD 686.95	
Pediatric Neurology Clinic	22 February 2024	24 February 2024	USD 2613.96	USD 181.94	USD 287.66	USD 230.99	USD 79,897.76
Pediatric Neurology Clinic	7 March 2024	9 March 2024	USD 2619.26	USD 178.19	USD 281.72	USD 230.99	USD 79,897.76
Pediatric Neurology Clinic (1 day) and Obstetrics and Gynecology Clinic (5 days)	24 March 2024	30 March 2024	USD 6784.18	USD 405.04	USD 586.16	USD 2616.02	USD 79,897.76
**Total Hospitalization Costs**			**USD 14,779.96**	**USD 897.94**	**USD 1343.61**	**USD 3764.96**	**USD 239,693.27**
**Overall Costs for Diagnosis, Treatment, and Medication**			**USD 15,796.37**	**USD 937.74**	**USD 1358.85**	**USD 4646.07**	**USD 239,693.27**

## Data Availability

The raw data supporting the conclusions of this article will be made available by the authors on request.
